# Steroid-Responsive Intraocular Lens Deposits: A Case-Based Review of Diagnosis and Management

**DOI:** 10.7759/cureus.82878

**Published:** 2025-04-23

**Authors:** Aiad Al-Essa

**Affiliations:** 1 Department of Ophthalmology, Maharishi Markandeshwar University, Solan, IND

**Keywords:** biocompatibility, corticosteroids, delayed-onset inflammation, intraocular lens (iol) deposits, uveitis

## Abstract

The standard surgical procedure for treating cataracts uses intraocular lens (IOL) implants that produce consistent results. Among millions of global cases annually, postoperative complications involving IOL surface deposits occur in less than 1% of patients. The diagnostic and therapeutic challenges in delayed-onset sterile IOL precipitates remain significant because these complications are both uncommon and present atypically. The presented review examines an unusual case where fibrotic gray-white deposits formed on the anterior IOL surface eight months post-surgery without any intraocular inflammatory indicators. Visual acuity declined from the condition until topical corticosteroids provided a dramatic response, which proved the sterile inflammatory origin of the condition. The presented case establishes a base for discussing the pathophysiology, classification, differential diagnosis, and management of IOL surface deposits. The proper diagnosis of IOL surface deposits requires clinicians to differentiate them from genuine lens opacification as well as from both infectious endophthalmitis and toxic anterior segment syndrome (TASS) because incorrect diagnoses could lead to unnecessary surgical procedures. The review discusses how patients with previous uveitis experience clinical effects and investigates the predictive power of statistical and AI-based models for identifying at-risk cases, as well as future needs for IOL design and postoperative imaging research. The appropriate management of sterile IOL deposits remains essential to protect vision and prevent invasive interventions, particularly for patients who have complex inflammatory backgrounds.

## Introduction and background

Postoperative intraocular inflammation is one of the most common complications after cataract surgery, even though this operation has high success rates around the world. Cataract surgery is performed over 20 million times a year worldwide, with good outcomes in most patients, but mild postoperative inflammation occurs in up to 90%-95% of cases. Despite the advances in surgical techniques like manual small incision cataract surgery and phacoemulsification, inflammation is an expected outcome of the healing process of cataract surgery. Topical anti-inflammatory drugs are usually effective in controlling inflammation in patients [1].

Fortunately, parents of people who wear intraocular lenses (IOLs) to correct myopia will attest to the amazing help they provide, as they can restore vision. However, foreign material is introduced into the human eye in the form of a glass lens, which can provoke unpredictable immune reactions, especially in patients with preexisting inflammatory ocular or systemic conditions. IOL surface deposits are among the rare inflammatory complications, occurring in less than 1% of cases, and even less than 0.05% of cases are due to delayed sterile deposits. These deposits are a distinct postoperative inflammatory condition with unique clinical management needs.

Inflammation early postoperatively may result in deposits of inflammatory cells, proteins, and other substances. Nevertheless, IOL surface deposits that occur months after surgery are rare and usually do not cause intraocular inflammatory signs like anterior chamber cells, flare, vitritis, or chorioretinal activity. Due to their atypical presentations, these diagnoses can be very difficult to make because they can resemble TASS or other processes such as infectious endophthalmitis or true IOL opacification. Because the symptoms of some immune-mediated syndromes and inflammatory mimickers often overlap, they may be misdiagnosed or the diagnosis may be uncertain [2]. Diagnostic complexity is increased in patients with granulomatous disease, infectious uveitis, or autoimmune syndromes, where immune interactions between systemic and ocular compartments may occur. Disruption of ocular immune privilege during eye surgery through tissue damage can evoke delayed or exaggerated responses of the immune system to implanted devices [3]. In some cases, the only symptom may be visual disturbances as the changes in IOL clarity develop without evidence of active inflammation.

Differentiation between surface deposits and changes in the material of the IOL itself is required. True IOL opacification is a structural change and is usually irreversible, while sterile deposits are often reversible with medical therapy. It is important to accurately identify unnecessary surgical interventions that may be avoided. Slit lamp biomicroscopy along with patient history and advanced imaging modalities like anterior segment optical coherence tomography (AS-OCT) or Scheimpflug imaging is needed to differentiate the different types of deposits (calcific, inflammatory, glistening, and silicone oil contamination) [4,5]. Postoperative outcomes are highly dependent on the material properties of IOLs. For instance, hydrophilic acrylic lenses are more susceptible to calcification than hydrophobic ones. Although studies of biocompatibility have helped to elucidate IOL behavior, unpredictable interactions still present a concern in eyes with underlying pathology [6]. Postoperative inflammation control remains the cornerstone of corticosteroids. There is ongoing debate about the comparative effectiveness of corticosteroid versus non-steroidal anti-inflammatory drug (NSAID) monotherapy or their combination for subclinical inflammation and visual outcome optimization [7].

Although cataract and IOL technology have improved, there are few studies of late-onset sterile deposits without overt inflammation. Often, there is no history, or these cases are carried off as cancers of elderly or immunocompromised patients [8]. Early visual demands may help detect such changes in pediatric and refractive populations [9]. However, the efficacy of such drug delivery systems, sustained-release corticosteroids, and novel anti-inflammatory agents in treating sterile deposits is still unclear [10].

Objective

This review aims to explore the rare yet clinically significant phenomenon of delayed-onset sterile IOL deposits, which can arise in the absence of classical signs of intraocular inflammation. Using a recently documented case as the basis for discussion, this review seeks to deepen understanding of the pathophysiological mechanisms and classification of IOL deposits, particularly in non-infectious presentations. Additionally, it discusses the challenges in differential diagnosis when active inflammation is not evident, evaluates the therapeutic efficacy of topical corticosteroids, and underscores the practical implications for ophthalmic surgeons in managing such atypical postoperative presentations. Furthermore, the review proposes future directions for research, including the development of diagnostic models and the innovation of IOL biomaterials with improved immunologic compatibility. Overall, the goal is to bridge the existing knowledge gap in the diagnosis and treatment of unusual IOL-related complications and to advocate for conservative, steroid-based management strategies before resorting to invasive surgical interventions.

## Review

Understanding IOL deposits: mechanisms, classifications, and risk factors

Surface deposits on IOLs develop through multiple factors that include both patient immune system reactions and IOL substance composition and surgical techniques, as shown in Table 1. After cataract surgery, the eye recognizes the implanted IOL as a foreign object that triggers an immune response, mainly in individuals with susceptible immune systems. Inflammatory cells such as macrophages, along with lymphocytes and multinucleated giant cells, enter the anterior chamber when the blood-aqueous barrier becomes disrupted during this immune response. The cells that attach to the IOL anterior surface create deposits that doctors might mistake for signs of infection or degeneration [11].

**Table 1:** Classification of IOL surface deposits based on composition and origin IOL: intraocular lens

Type of deposit	Origin and contributing factors	Clinical characteristics	References
Glistenings	Seen in hydrophobic acrylic lenses and related to water microvacuole formation	Causes light scatter and reduced contrast sensitivity, with the lens remaining structurally intact	[5]
Calcific deposits	Common in hydrophilic acrylic lenses and associated with lens material degradation	Irreversible calcium-phosphate crystal deposition typically requires IOL explantation	[6]
Inflammatory cell deposits	History of uveitis and postoperative inflammation	Composed of immune cells (e.g., macrophages, lymphocytes) and reversible with anti-inflammatory therapy	[12]
Silicone oil contamination	Occurs after vitreoretinal surgeries involving silicone oil use	Silicone droplets adhere to IOL surface and can mimic inflammatory or fibrotic patterns	[13]

**Figure 1:**
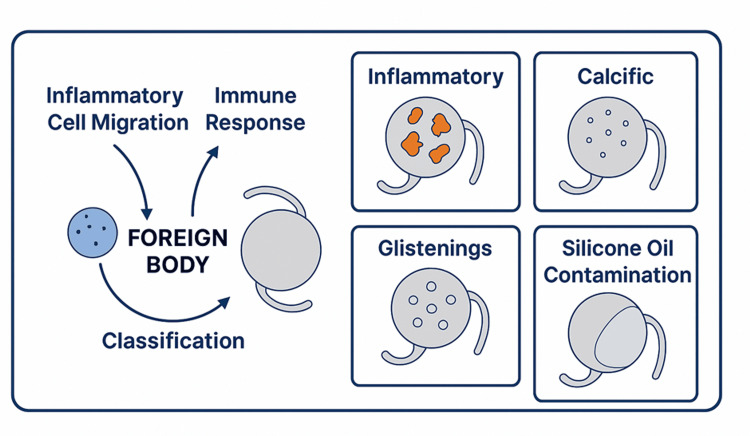
Pathophysiology and classification of IOL deposits IOL: intraocular lens The image is created by Aiad Al-Essa.

The classification system assists in medical diagnosis while revealing the biological processes between tissue and materials, supporting the development of treatment plans for both conservative and surgical interventions (Figure 1).

Several factors related to both patients and procedures enhance the risk of IOL surface deposits forming. The development of intraocular inflammation from chronic or recurrent uveitis functions as the main risk factor. The immune response to intraocular devices remains elevated following clinical resolution of toxoplasmosis-induced uveitis, according to research [14]. IOL materials have different susceptibilities to calcification, with hydrophilic lenses being more prone, while hydrophobic polymers attract fewer immune cells but are still prone to glistening formation. Surgical factors also contribute significantly. The development of postoperative complications depends on three surgical elements: the duration of intraocular manipulation, the remaining lens epithelial cells, and the selection and amount of viscoelastic substances employed during surgery. Postoperative inflammation becomes worse when cortical cleanup is incomplete, when the posterior capsule ruptures, or when surgeons handle the iris excessively because these conditions increase the risk of deposit formation [15]. The accurate diagnosis and effective preventive strategies, alongside appropriate management of IOL-related complications, depend on understanding all three dimensions of factors, including mechanical aspects, material composition, and surgical procedures, especially when treating complex or high-risk patients. Medical practitioners can use early diagnosis to differentiate between treatable inflammatory deposits and permanent IOL changes, which enables them to choose between medical interventions and surgical procedures.

Late-onset sterile deposits and diagnostic considerations

Sterile deposits that appear on IOLs after cataract surgery manifest late in the postoperative period at rates considered extremely rare and difficult to diagnose. These deposits emerge several months after unproblematic cataract surgery when no intraocular inflammatory signs are present. Delayed cases of sterile deposits show symptoms of blurred vision, along with glare and reduced contrast sensitivity, instead of anterior chamber cells and flare, as well as vitritis, which are typically observed in early postoperative reactions. These deposits possess significant clinical importance because they produce meaningful visual impairment that imitates serious postoperative conditions [16]. These particular cases stand apart because they remain without clinical signs and develop late after surgery. The late-onset changes differ from early inflammatory deposits since they develop in quiet eyes without anterior segment reactions, posterior segment activity or systemic symptoms. The diagnosis becomes challenging when IOLs exhibit a hazy appearance or show gray-white deposits because patients experience visual decline that cannot be explained by posterior capsular opacification or tear film instability. Medical practitioners should maintain elevated levels of suspicion regarding sterile inflammatory deposits when treating patients who have a history of ocular inflammation or systemic autoimmune disease [17].

Accurate identification of sterile IOL deposits requires differentiation from other possible conditions, including true IOL opacification, infectious endophthalmitis, and toxic anterior segment syndrome (TASS), because their management strategies differ substantially. True IOL opacification, which occurs from calcification or surface degradation, becomes permanent and leads to the need for lens explantation or exchange. The treatment and diagnostic value of topical corticosteroids prove effective for sterile inflammatory deposits in the eyes. Endophthalmitis from infections remains rare in delayed cases but triggers similar symptoms, including pain, together with redness and hypopyon formation, and systemic manifestations, which demand immediate medical or surgical intervention with antibiotics or vitrectomy. TASS develops as a non-infectious condition between 12-48 hours after surgery while causing diffuse inflammation, elevated intraocular pressure, and damage to endothelial cells to differentiate it from other late sterile presentations [18]. The diagnostic process depends on a complete clinical investigation and targeted examination of the eye because inflammatory indicators cannot be identified. Previous episodes of uveitis that went into remission should trigger suspicion about IOL-induced immune reactions. The choice between hydrophilic and hydrophobic acrylic IOL materials matters for diagnosis because hydrophilic materials show higher susceptibility to calcification and immune deposition. The surgical procedures, which involve posterior capsule rupture, retained lens material, and excessive iris manipulation, can delay the immune response.

The slit-lamp biomicroscope serves as the primary tool for observing both the appearance and placement of deposits. The distinction between deposits and actual lens degradation becomes possible through the identification of fine granular or plaque-like material, along with central or peripheral distribution or adherence to the anterior surface. Uncertain cases require additional imaging using AS-OCT or Scheimpflug imaging to determine whether deposits exist on the surface or inside the optic material, or if they are caused by capsular opacification [19,20]. Medical staff who identify these benign deposits early can administer topical corticosteroids for treatment, which avoids unnecessary surgical procedures. The correct diagnosis of these underreported deposits is essential since misdiagnosis results in either unnecessary medical treatment or delayed appropriate therapy, thus requiring precise clinical diagnosis and awareness from medical professionals.

Therapeutic insights: role of steroids and case-based perspective

Delayed-onset, sterile deposits on IOLs are a rare but clinically important postoperative complication associated particularly with patients suffering from ocular inflammation or with immune predisposition. Epidemiology of nodular corneal dystrophy akin to the nodulare specsiäse kataractae reports that these deposits can occur months after cataract surgery, lacking any history of inflammation, and the presence of the deposits does not specifically rule out previous uncomplicated cataract surgery. With the appropriate treatment, visual prognosis is excellent in this setting; therefore, differentiating such deposits from other causes of postoperative visual decline (such as infectious endophthalmitis, TASS, or irreversible IOL opacification) is essential, given marked differences in treatment. Case-based evidence supported by clinical experience suggests that a trial of corticosteroid therapy provides therapeutic as well as diagnostic insight in these circumstances.

The first case was a 41-year-old male who had toxoplasmosis chorioretinitis and who had phacoemulsification with IOL implantation in his right eye. It had an uneventful immediate postoperative course, and no steroidal therapy was required. However, he had a gradual visual decline without ocular discomfort or systemic symptoms, occurring eight months later. Fibrotic gray-white deposits were seen on slit lamp biomicroscopy on the anterior surface of the IOL optic. There were no anterior chamber cells, flare, vitritis, or chorioretinal lesions on anterior and posterior segment examinations, which were otherwise unremarkable for evidence of inflammation. There were no inflammatory indicators present; as such, the medical team took a conservative approach and prescribed topical prednisolone acetate 1% four times daily. After several weeks, the patient had markedly improved visual clarity and resolution of the deposits. Consequently, this outcome confirmed that the deposits were of an inflammatory and sterile nature, and that corticosteroid responsiveness presents therapeutic and diagnostic value in such settings [21].

The variation of persistence and evolution of IOL surface deposits without treatment is significant. However, some deposits may stay stable or clear spontaneously; others may progress and possibly result in visual impairment that prompts medical treatment [22]. Uveitic history or uveitic patients are still treated with corticosteroids as the mainstay of treatment for postoperative inflammatory conditions. Both systemic and local corticosteroids have been validated by the Multicenter Uveitis Steroid Treatment (MUST) Trial as achieving the role of preserving vision and minimizing complications in these patients [23]. A second case was adapted from published literature and concerned a 56-year-old woman with a history of idiopathic uveitis who had undergone cataract surgery with implantation of a hydrophobic acrylic IOL (Acrysof MA60BM) in her left eye. She was seen six months postoperatively with mild glare and visual blurring. The anterior IOL surface was visible on slit-lamp examination with superficial white deposits, with no evidence of uveitic reactivation or intraocular inflammation. The presence of hyperreflective surface material was confirmed by AS-OCT. Following a diagnostic therapeutic trial with prednisolone acetate 1% four times daily, the deposits were nearly completely resolved, and the vision recovered uneventfully within three weeks. In the context of reported steroid responsiveness, emphasized in this case, the value of steroid responsiveness as a non-invasive way to diagnose subclinical inflammation, especially in eyes immunologically predestined, is further highlighted [24].

Third is a case, accompanied by Werner and colleagues, of a 68-year-old man with a prior pars plana vitrectomy who underwent phacoemulsification with hydrophilic acrylic IOL implantation. Nineteen months later, he returned presenting with hazy vision and decreased contrast sensitivity, at the time of hazy vision, and without ocular pain or redness. Fine granular gray-white deposits on the surface of the anterior IOL could be seen with slit lamp examination. Deep deposition and structural compromise of the lens were confirmed by imaging with AS-OCT. The patient was initially treated empirically with topical antibiotics and corticosteroids, then treated with corticosteroid monotherapy after exclusion of infection. The deposits resolved over the following month, and vision returned to baseline. Furthermore, this case illustrates the vulnerability of hydrophilic lenses to decreased immune protection and reacts to highlight the beneficial role of first-line conservative management, using topical corticosteroids, for early management [25].

In each scenario, there is a common thread that runs through the patient's clinical history: patients have made use of topical corticosteroids for the treatment of delayed-onset IOL deposits regardless of the type of IOL being used, the patient's history, or the particular surgical setting. The inflammatory nature of the deposits was confirmed by the therapeutic response, which prevented unnecessary surgical procedures. Notably, these responses also help to distinguish these deposits from those snowballs, which are often irreversible as lesions like calcification and glistenings, which tend not to resolve normally with medical therapy and may require explantation [26,27].

One of the diagnostic difficulties in such cases is to distinguish reversible immune-mediated deposition from true material degradation. Hydrophilic acrylic lenses are more susceptible to calcific deposition, and hydrophobic lenses are less susceptible but not immune to sterile inflammatory reactions. Therefore, an accurate diagnosis necessitates a careful awareness of IOL biomaterials, ocular history, slit lamp biomicroscopy, and confirmatory imaging.

**Figure 2:**
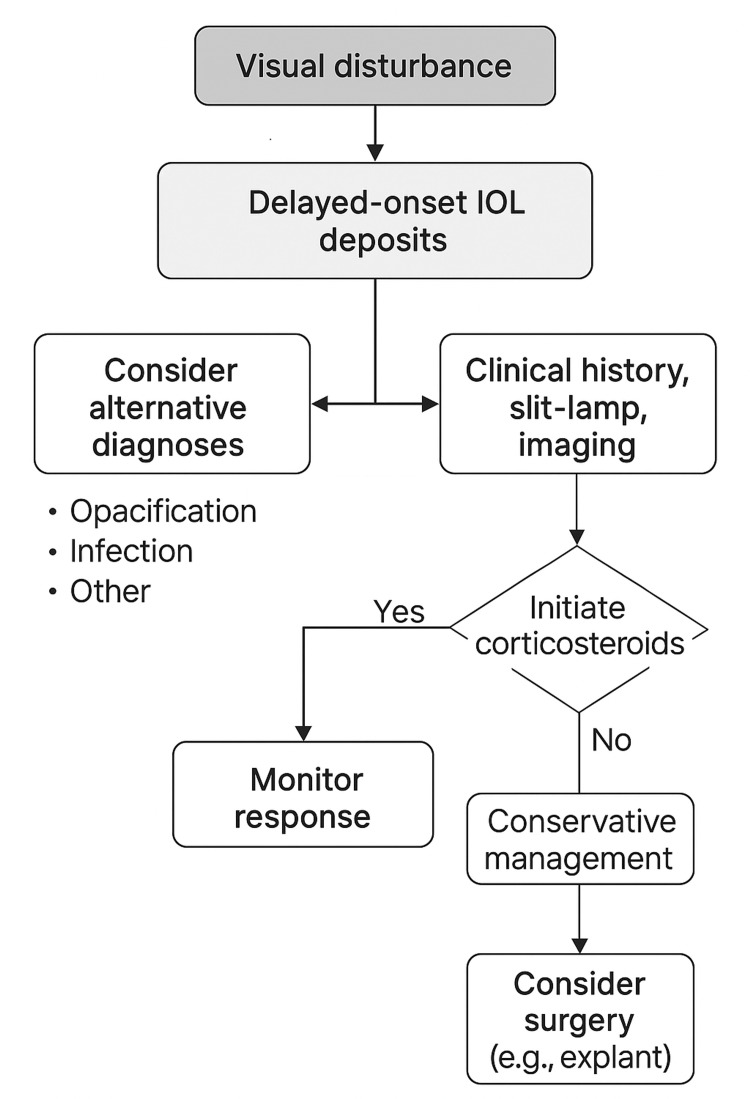
Management algorithm for delayed-onset IOL deposits IOL: intraocular lens The image is created by Aiad Al-Essa.

The clinical safety and information gained from a short diagnostic corticosteroid trial are especially safe and informative in the situation of unexplained visual deterioration without overt signs of infection or inflammation. However, when these tools are used in conjunction with imaging tools like AS-OCT or Scheimpflug photography, this approach becomes a high-yield, low-risk strategy for restoring vision with minimal or no recourse to surgical procedures. An approach to implementing this process is developed, and a clinical management algorithm is presented in Figure 2.

Clinical implications, modeling considerations, and future directions

Clinical Implications in High-Risk Patients

The review focuses on a unique but important clinical situation where sterile deposits appear on IOLs months after cataract surgery in patients with controlled uveitis. The appearance of these deposits occurred without active anterior or posterior segment inflammation, while early postoperative inflammation would typically appear within days to weeks, and standard treatment plans work effectively for its management. These deposits appeared as gray-white fibrotic tissue, which was visually significant but did not present with uveitic reactivation or signs of infection. The clinical presentation makes it difficult for doctors to identify non-harmful visual findings from surgically necessary pathologies [27]. Topical application of prednisolone acetate 1% produced swift positive results by clearing the deposits and improving vision in a short amount of time. The observed corticosteroid response validates that these inflammatory deposits were non-infectious and subclinical while supporting the use of corticosteroids in such cases through a conservative therapeutic trial. The condition of permanent IOL opacification from material degradation or calcification cannot be treated with medical interventions, so patients may need surgical removal or new lens implantation [28]. A diagnostic and therapeutic steroid trial at the beginning of treatment serves dual clinical and prognostic purposes because it precedes invasive procedures when uveitis or endophthalmitis symptoms are absent.

This particular case strengthens the need for personalized medical care among patients who have uveitis, autoimmune diseases, or systemic inflammatory conditions. The immune system of these patients functions subtly to react against foreign objects, including IOLs, even after their surgical placement [29]. The surface usually looks quiet to clinical assessment, yet subtle inflammatory activities or immune monitoring processes might persist, which leads to delayed manifestations, including surface deposits. Medical treatment successfully reversed IOL deposits in patients with uveitic eyes according to recent case report findings [30]. The clinical practice demands that medical professionals handle visual quality changes with care, particularly for patients with complicated conditions or immunological susceptibilities. Timely identification of delayed inflammation without uveitis, together with specific diagnostic tests including slit-lamp biomicroscopy and AS-OCT, and brief corticosteroid treatment as a testing method, helps minimize unnecessary treatment and protects vision health. This case demonstrates why healthcare organizations must use collaborative care models that involve rheumatologists or immunologists for patients whose systemic factors affect their eye health outcomes. Table 2 summarizes key diagnostic and therapeutic decision points for various IOL deposit types, synthesizing onset timing, treatment responses, and prognostic indicators to assist in clinical decision-making.

**Table 2:** Diagnostic and therapeutic considerations for IOL deposits OCT: optical coherence tomography; IOL: intraocular lens

Deposit type	Typical onset	Key clinical features	Steroid responsiveness	Imaging findings	Management strategy	References
Inflammatory deposits	Early or delayed postoperative period	Gray-white anterior deposits and no inflammation	High	Anterior segment OCT shows superficial deposits	Topical corticosteroids first-line	[12],[21]
Calcific deposits	Usually delayed (months to years)	White crystalline opacity and irreversible	None	Dense hyperreflective surface changes	IOL explantation required	[6],[25],[26]
Glistenings	Gradual over time	Shiny vacuoles within the optic and visual glare	None	Internal optic vacuoles on the slit-lamp	Observation or IOL exchange if symptomatic	[5],[18]
Silicone oil contamination	Post-vitrectomy or oil use	Oily droplet appearance and irregular reflection	None	Droplet-like reflections on retroillumination	Observation or polishing, if feasible	[11],[25]
Pigment dispersion	Post-laser or trauma	Pigment granules on IOL surface	Low	Scattered high reflectivity foci	Anti-inflammatory drops; laser if persistent	[16],[2]
Fibrotic plaques	Chronic postoperative	Dense plaque over an optic zone	Variable	Capsular wrinkling and plaques	Capsulotomy or IOL exchange	[25],[26]
Proteinaceous coating	Postoperative inflammation	Hazy film reduces clarity	Moderate	Diffuse hyperreflective film	Steroids, possibly IOL polish	[10],[11]
Foreign body giant cells	Weeks to months	Multinucleated cell clusters	Low	Granular clumps on the IOL surface	Often surgical removal	[14],[15]
Biofilm formation	Post-surgery in infected cases	Sticky, irregular, and resistant to cleaning	None	Adherent film seen on imaging	Explanation + antimicrobial therapy	[28],[24]
Drug-induced deposits	After intracameral drug use	Diffuse haze and drug history linked	Variable	Diffuse surface haze	Discontinue the drug and supportive care	[7]

Mathematical and Predictive Modeling Considerations

The combination of data science and quantitative analytics with ophthalmology creates new pathways for personalized patient care that develop methods to detect and manage uncommon yet important problems. The diagnosis of delayed-onset sterile IOL deposits can benefit from predictive modeling because clinical detection remains challenging. The analysis using multivariate logistic regression should include patient age and uveitis history, as well as systemic autoimmune conditions and IOL material composition, postoperative steroid responsiveness, and visual acuity changes over time. Clinical analysis of multiple risk factors enables more accurate predictions about the potential development of deposits, thus aiding in strategic decision-making and customized care-based follow-up programs [31].

Survival analysis methods, including Kaplan-Meier curves and Cox proportional hazards models, should be used to determine the time needed for deposit formation and steroid therapy resolution outcomes. Combined data about deposit grading from slit-lamp examination, visual function scores, and treatment responses could help develop therapeutic models that improve intervention thresholds. Such tools find their best applications in clinical research while providing clinicians with the ability to manage patients according to personalized risk assessments. AI and machine learning systems show great potential to transform diagnostic procedures for imaging the anterior segment, and doctors anticipate this pivotal development. Large datasets of AS-OCT and fundus of Scheimpflug images succeed in training convolutional neural networks (CNNs) to locate subtle IOL surface irregularities that medical professionals may miss. These diagnostic tools would help medical staff detect sterile inflammatory deposits at an earlier stage while differentiating them from permanent lens opacification and fibrotic changes [32]. Through deep learning, AI systems would achieve two functions: they would detect deposits while simultaneously evaluating their severity and predicting therapeutic outcomes to enhance medical choices.

Mousedown point-of-care assistance becomes feasible with biometric input tools such as axial length and IOL power, which combine with surgical technique and clinical parameters through mobile platforms used by ophthalmologists. Digital health tools enabled by technology would notify at-risk patients beforehand or throughout their follow-up visits, thus enabling improved evaluations and aggressive monitoring [30]. The automation system would become essential to maintain standard surgical care alongside shortened diagnostic times and reduced need for additional surgical operations in high-volume procedures. AI prediction tools, along with mathematical models, can revolutionize sterile IOL management by transforming it into proactive care due to their ability to provide clinical prediction support in standard eye care procedures [24]. The administration of corticosteroids for deposit management in these cases produces potential adverse drug reactions, which include elevated intraocular pressure, delayed wound healing, and an increased risk of cataract formation, especially during extended treatment periods [33].

Future Directions and Research Priorities

The medical literature contains scattered reports about delayed-onset IOL surface deposits, yet systematic research on this topic remains insufficient on a large scale. The understanding of these deposits requires multi-institutional prospective case registries to establish their clinical behavior and scope. These databases will establish incidence statistics as well as patient risk factors and IOL material sensitivities, along with treatment results for both normal eyes and those affected by uveitis. The collected data will enable the development of evidence-based guidelines and diagnostic approaches to help medical professionals identify and manage these less common yet visually significant complications from the beginning. Research on biomaterials must progress simultaneously with the development of improved IOL designs. Future IOLs must achieve high optical quality while incorporating better biocompatible properties, especially designed to serve patients with inflammatory or autoimmune conditions. The future of IOL development depends on research that focuses on hydrophobic acrylic materials and surface treatments that decrease protein binding and drug-eluting lenses that release small amounts of anti-inflammatory agents. The new developments show promise to decrease postoperative immune reactions and eliminate the requirement for corticosteroid therapy through topical or systemic administration. Individualized patient care represents a fundamental element for future strategic planning. The reaction to delayed IOL deposits differs between patients, and so does their willingness to accept medical intervention. Patients who have one eye or experience high surgical fear and systemic illnesses usually avoid IOL removal procedures even when their vision quality deteriorates. When facing such situations, healthcare professionals need to deliver conservative treatments based on steroids while practicing shared medical decision-making. Patient education about reversible visual symptom causes alongside non-surgical treatment options builds trust, which enhances their commitment to treatment procedures [33].

The use of corticosteroids in pharmacological treatment shows effectiveness but presents specific limitations for patient care. The long-term utilization of these drugs, such as prednisolone acetate, dexamethasone, and fluorometholone, leads to established medical risks that include elevated intraocular pressure, delayed wound healing, and posterior subcapsular cataract development in people with phakic eyes, as well as general immune system suppression in patients needing long-term treatment. Studies of inflammatory diseases demonstrate the necessity for alternative ocular immunomodulators because current medications produce a systemic burden while maintaining anti-inflammatory effectiveness [34]. Management strategies continue to advance, which requires growing importance for interdisciplinary teamwork. The diagnosis and proper treatment of hidden eye symptoms in patients with systemic illnesses depends on communication between specialists, including rheumatologists and internists, and primary care providers [35]. These directions emphasize the necessity of implementing a comprehensive approach to preventing and treating sterile IOL surface deposits, which requires clinical monitoring, material improvements, patient-focused treatment, and evolving pharmacological approaches.

## Conclusions

The evaluation was of sterile deposits that form on IOL surfaces after uneventful cataract surgery and are not accompanied by signs of inflammation. To demonstrate that these visible deposits react positively to topical corticosteroid treatment, the article analyzed three different case examples. All three cases of patients with toxoplasmosis chorioretinitis, controlled idiopathic uveitis, and vitrectomy history responded positively to topical corticosteroid therapy without surgical procedures. These are immune-mediated, but subclinical inflammatory reactions that are not due to IOL material degeneration or infection, and can be successfully treated with steroids in these three cases. This condition can be correctly identified so that surgeons do not perform unnecessary procedures, such as IOL explantation, in patients who already have inflammatory risks.

Patient-specific care that combines historical background assessment with non-invasive imaging is key for the early discovery of these health conditions. When no infection or over-inflammation exists, the detection of such deposits should prompt a diagnostic and therapeutic trial with topical corticosteroids as the primary treatment approach. The vision outcomes from this risk-averse treatment approach are improved, while operational risks are lower. Work on predictive models, AI imaging systems, and IOL biomaterial development will be important for future investigations aimed at preventing more incidents and achieving better diagnostic results. Ophthalmic providers will achieve patient-centered management of late-onset IOL-related complications through collaborative care models supported by data-driven strategies.
